# Electrodialysis Deacidification of Acid Hydrolysate in Hemicellulose Saccharification Process: Membrane Fouling Identification and Mechanisms

**DOI:** 10.3390/membranes13030256

**Published:** 2023-02-21

**Authors:** Xitao Luo, Lingling Sun, Qinghui Shou, Xiangfeng Liang, Huizhou Liu

**Affiliations:** 1 CAS Key Laboratory of Bio-Based Materials, Qingdao Institute of Bioenergy and Bioprocess Technology (QIBEBT), Chinese Academy of Sciences (CAS), Qingdao 266101, China; 2 University of Chinese Academy of Sciences, Beijing 100049, China

**Keywords:** hemicelluloses, saccharification, deacidification, electrodialysis, membrane fouling

## Abstract

Acid saccharification of hemicelluloses offers promising pathways to sustainably diversify the revenue of the lignocellulose biorefinery industry. Electrodialysis to separate inorganic acids from acid hydrolysate in the hemicellulose saccharification process could realize the recovery of sulfuric acid, and significantly reduced the chemical consumption than the traditional ion exchange resins method. In this work, the deacidification of corncob acid hydrolysate was conducted by a homemade electrodialysis apparatus. The results showed that: (1) more than 99% of acid can be removed through the electrodialysis process; (2) A non-negligible membrane fouling occurred during the electrodialysis process, which aggravated with the repeated batch running The final global system resistance rose from 15.8 Ω (1st batch) to 43.9 Ω (10th batch), and the treatment ending time was delayed from 120 min (1st batch) to 162 min (10th batch); (4) About 90% of protein, 70% of ferulate acid, and 80% of *p*-coumarate acid precipitated from the corncob acid hydrolysate during the electrodialysis process. The zeta potential of corncob acid hydrolysate changed from a positive value to a negative value, and an isoelectric point around pH 2.3 was reached. HSQC, FTTR, and GPC, along with *SEM* and *EDS* analysis, revealed that the fouling layers mostly consisted of hydrolysates of protein and lignin. The result of HSQC indicated that the membrane foulant may exist in the form of lignin–carbohydrate complexes, as the lignin component of the membrane foulant is in the form of *p*-coumarate and ferulate. From the result of FTIR, a strong chemical bonding, such as a covalent linkage, existed between the lignin and protein in the membrane foulant. Throughout the electrodialysis process, the increased pH decreased the stability of colloidal particles, including lignin and proteins. Destabilized colloidal particles started to self-aggregate and form deposits on the anion exchange membrane’s surface. Over time, these deposits covered the entire membrane surface and the spaces between the membranes. Eventually, they attached to the surface of the cation exchange membrane. In the end, a suggestion to control and minimize membrane fouling in this process was discussed: lower pH as a process endpoint and a post-treatment method.

## 1. Introduction

In terms of lignocellulose valorization, the “hemicelluloses-first strategy” is a significant and valuable prior-fractionation method to separate the lignocellulose component (cellulose, hemicellulose, and lignin) in the plant cell wall [1,2]. Hemicelluloses, depending on their characteristic of being highly thermo- and acid-liable, are easy to degrade to a pentose (xylose, arabinose, etc.) or oligosaccharides by the acid hydrolysis method (acid saccharification) or by enzymatic hydrolysis. Enzymatic hydrolysis has been reported to degrade hemicellulose to xylose, but it is costly and immature and has not yet been adopted by major companies worldwide [3]. Dilute sulfuric acid saccharification is still a dominant industry pattern to produce pentose by hard wood or herbage [4,5]. However, in the traditional acid saccharification process, the main drawbacks are the costs and environmental overloading problems of an ion-exchange unit for deacidification. Ion-exchange resins are so overly dependent and inefficient that they limit the development of saccharification. Therefore, a novel process that is environmentally friendly, has a low cost, and has a high yield is highly expected. 

Recently, novel green technologies, such as membrane filtration, lime treatment, and chromatographic separation, were applied in relevant saccharification processes [6,7,8,9,10,11]. Among them, the electrodialysis (ED) process, which could selectively separate ions by using an ion-exchange membrane driven by an electric field, is the most promising. Lemaire et al. originally developed a purification process combing ultrafiltration (UF), ED, and ion-exchange resin to purify pentose [12,13]. UF is operated as a pretreatment unit to eliminate hazardous macromolecules. More than 80% of sulfuric acid could be recovered from monosaccharides without chemical consumption or waste generation. Meanwhile, the energy consumption and loss ratio of sugar are within an acceptable range. However, in these studies, the influence of lignin and protein as membrane foulants was not clear.

Membrane fouling is an intrinsic problem of the ion exchange membrane during ED operations. A full understanding of fouling identification and mechanisms is vitally important to the membrane’s life and performance. Dammak et al. put forward a systematical discussion on the ion-exchange membrane’s fouling during the electrodialysis process, including the fouling types, influences on the process, characterization methods, fouling mechanisms, cleaning methods, and so on. Proteins, and polyphenols, as the typical membrane foulant, were analyzed for the fouling mechanisms. Moreover, modern non-destructive membrane cleaning methods are discussed [14,15]. Haddad et al. extracted lignin from Kraft black liquor through ED. The fouling involves several steps: the destabilization of lignin macromolecules through promoted protonation with pH decrease, the formation of lignin clusters on the surface of bipolar membranes, and the attachment of lignin clusters to cationic membranes after the saturated adsorption on bipolar membranes. Based on this conclusion, the pulsed electric field method was introduced in their work. And a chemical cleaning strategy using caustic soda and freshly diluted black liquor as cleaning solutions was tested [16,17,18]. To the best of our knowledge, there has been no report regarding the behavior when the ED is operated as a deacidification unit of the acid hydrolysate in the hemicellulose saccharification process.

In this work, deacidification of the hemicellulose saccharification process was carried out through an ED operation. The aim of this work is to (1) identify the nature of the membrane foulant and (2) investigate the mechanisms of particle deposit on the surface of the membrane during the electrochemical deacidification process in order to control and eventually minimize this process drawback. Furthermore, based on our understanding of this investigation, we wish to propose proper configurations and cleaning methods to prevent and/or minimize this process obstacle.

## 2. Materials and Methods

### 2.1. Materials

Corncob acid hydrolysate (CAH) was kindly provided by Shandong Futaste Group, China. The hydrolysis reaction was conducted at 120–125 °C for 120 min with a 1.0 % (*w*/*w*) dosage of dilute sulphuric acid and a liquid-to-solid ratio (L/S) of 8:1. The corncob was pre-washed in hot water to remove the ash and impurities.

The main components of CAH are listed in Table 1. Analytical-grade chemicals, including sulfuric acid, and sodium sulfate, were purchased from Sinopharm, China. All the chemicals were used as received.

### 2.2. Electrochemical Deacidification Apparatus and Protocol

**Figure 1:**
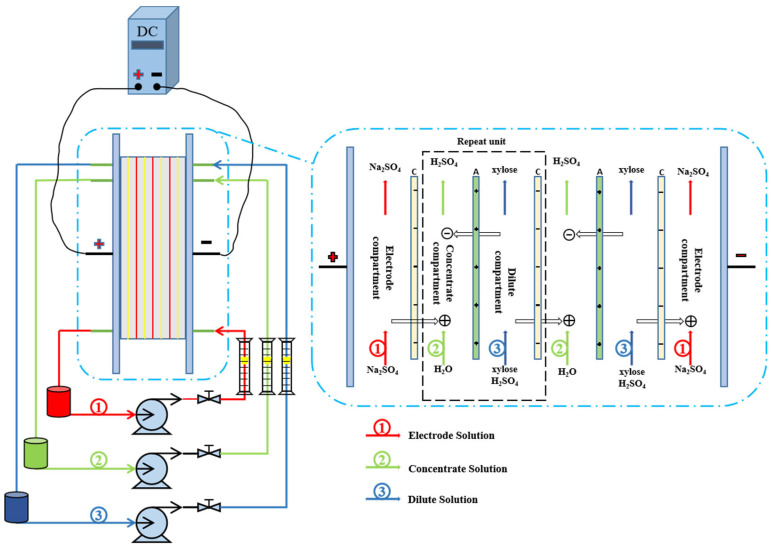
Schematic representation of the ED applied to electrochemical deacidification of CAH. C: CEM, A: AEM.

The ED stack was composed of dilute compartments (for CAH), concentrate compartments (for pure water), and two electrode compartments on each side (for Na_2_SO_4_ solution). 5 pieces of cation exchange membranes (CEM) and 4 pieces of anion exchange membranes (AEM) were placed alternately to make up 4 repeating units of the membrane stack (Figure 1). The active surface area of each membrane was 186.55 cm^2^ (9.1 × 20.5 cm). The anode and cathode were made of titanium coated with ruthenium. The whole ED stack, including the AEMs and CEMs, was from Shandong Tianwei Membrane Technology Co., Ltd. (Weifang, China). The properties of these membranes are shown in Table 2. Three pumps were set up to circulate the solutions from their reservoir (with magnetic stirring) to the stack. The constant current between two electrodes was provided by a DC power supply (model: LongWei LW-K605D, Hong Kong). A jacket coil heat exchanger was installed in each reservoir to maintain a constant temperature. 

All the experiments were performed in repeated batch mode using 2 L of CAH (0.22 μm filtered), 2 L of pure water, and 1 L of Na_2_SO_4_ (0.1 mol/L). The process was stopped when the electrical conductivity reached 1000–1100 μS/cm. Ten repeated batches of the electrochemical deacidification experiment were performed without any treatment. The main process conditions are summarized in Table 3. The applied current, voltage variation, electrical conductivity, pH, and temperature of each reservoir were recorded every 18 min in the first 120 min. After 120 min, the data were recorded every 6 min. 

### 2.3. Process Evaluation

#### 2.3.1. Global System Resistance

The global system resistance was computed by recording the applied current and voltage variation along the ED process and employing Ohm’s law:

R=U/I

where *R* is the global system resistance (Ω), *I* is the applied current (A), and *U* represents the voltage across the ED stack (V) (Bazinet et al., 2000; Haddad et al., 2017a).

#### 2.3.2. Deacidification Ratio

The deacidification ratio during the ED process was calculated by the following equations:

$$Deacidification\;ratio\;\left(\%\right)=\frac{V_{i}C_{i}-V_{f}C_{f}}{V_{i}C_{i}}\times 100\%$$

Cx=10^−pHx

where *V_f_ C_f_* and *V_i_ C_i_* respectively are the final and initial moles of the H^+^ in the solution, *x* means the *i* or *f*, respectively. 

The pH was measured by a pH meter fitted with a temperature compensator. It is assumed that the activity coefficients were equal to 1 in this process. 

#### 2.3.3. H^+^ Relative Energy Consumption

The performance of an ED process can be evaluated by determining the energy consumption of the system [19]. In this work, the relative energy consumed in each ED batch was calculated by the following equation:

$$E_{H}=\frac{I\int_{t_{i}}^{t_{f}}Udt}{3600\left(V_{i}C_{i}-V_{f}C_{f}\right)}$$

Here, *E_H_* is the relative energy consumption (Wh) per mole of H^+^ production, and *t* shows the duration of the ED operation (s). Note that the consumed energy of the pumps and hot water bath was not included in the *E_H_* calculation.

### 2.4. Analysis Methods

#### 2.4.1. Quantitative Analysis of Monosaccharides and Phenolic Acids

The content of monosaccharides in CAH (0.22 μm filtered) was measured by an HPLC system (Agilent Model 1200, Agilent Technologies Inc., Santa Clara, CA, USA) equipped with a Bio-Rad Aminex HPX-87H column (300 mm × 7.8 mm) and refractive index detector. The column was operated at 55 °C with a 0.005 M/L H_2_SO_4_ solution as the mobile phase at a flow rate of 0.6 mL/min. 

#### 2.4.2. pH Value and Conductivity of CAH

A conductivity meter (range: 20–199.9 ms/cm, P902, Shanghai Youke Instrument Co., Ltd., Shanghai, China) and pH meter (P901, Shanghai Youke Instrument Co., Ltd., Shanghai, China) were used to measure the electrical conductivity and pH of the samples at each stage of the experiment, respectively.

#### 2.4.3. Quantitative Analysis of Protein in CAH

Protein concentration was determined using the Bradford method (Bradford, 1976) with a Bradford protein assay kit (Beyotime, Shanghai, China). Different concentrations (0.2, 0.4, 0.6, 0.8, 1.0, and 1.2 g/L) of bovine serum albumin (BSA; Sangon Biotech, Shanghai, China) were prepared as standards. The absorbance of samples at 595 nm in a 96-well plate was measured using a microplate reader (Aosheng AMR-100, Hangzhou, China).

#### 2.4.4. Zeta Potential of Colloidal Particles in CAH

The zeta potentials of the CAH during the ED process were determined with a Zetasizer Nano ZSP (Malvern Instruments, Worcestershire, UK). The CAH was filtered by an ultrafiltration membrane (0.22 μm) before measurement.

#### 2.4.5. Electron Microscopy and Elemental Analysis

The samples were subjected to observation by using a scanning electron microscope (SEM, Hitachi S-4800, Tokyo, Japan) at 5.0 kV. The energy dispersive X-ray spectroscopy (EDS) conditions were a 5 kV accelerating voltage with a 15 mm working distance and 250× magnification. The EDS analysis provides a relative percentage of the surface elemental compositions. Prior to the SEM and EDS analyses, vacuum-dried samples were coated with a thin layer of gold to improve the image quality.

#### 2.4.6. Determination of Molecular Weight

The molecular weights of the membrane foulant were determined by gel permeation chromatography (GPC) on a PL gel Olexis column (300 × 7.5 mm, Polymer Laboratories Ltd, Amherst, MA, USA), calibrated with pollutant polysaccharide standards. A flow rate of 1.0 mL/min was maintained. The eluent was DMSO. Detection was achieved with a Knauer differential refractive index detector (RID). The column oven was kept at 40 °C. Samples were dissolved in DMSO at a concentration of 0.2%.

#### 2.4.7. FTIR (Fourier Transform Infrared Spectrometer)

FTIR analyses of the samples were carried out on a Thermo Nicolet FTIR spectrometer (Nicolet 6700, Thermo Fisher Scientific, Inc., Waltham, MA, USA) in the wavenumber range of 400–4000 cm^−1^ with a resolution of 4 cm^−1^. Before analysis, the samples were freeze-dried to obtain a dry powder. The powders were then ground with approximately 200 mg of KBr and pressed into a pellet. 

#### 2.4.8. HSQC (Heteronuclear Single Quantum Coherence)

Two-dimensional HSQC (2D-HSQC) spectra were recorded on a Bruker AVANCE-III 600 MHz spectrometer at 25 °C. Before analysis, the samples were freeze-dried, and then 20 mg was dissolved in 0.5 mL of dimethyl sulfoxide-d6 (DMSO-d6).

## 3. Results and Discussion

### 3.1. Evaluation of ED Parameters

In this work, ED process parameters, including pH, global system resistance, deacidification ratio, and energy consumption were analyzed, in order to evaluate the process efficiency and the extent of membrane fouling.

#### 3.1.1. Global System Resistance

**Figure 2:**
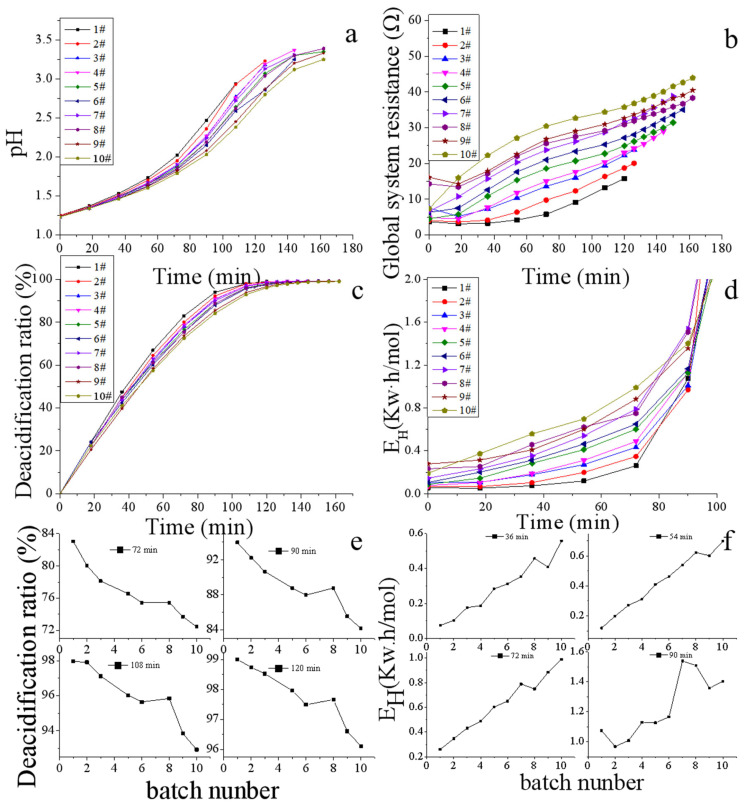
Evaluation parameters of ED system during the electrochemical deacidification of CAH. (**a**) pH, (**b**) global system resistance, (**c**) deacidification ratio, (**d**) H^+^ relative energy consumption, (**e**) instantaneous deacidification rate, (**f**) instantaneous H^+^ relative energy consumption.

With the deacidification by the ED process, the pH value was increased gradually (Figure 2a). Figure 2b shows the plots of the global system resistance throughout the ED process in the 10 consecutive batches. Along with the 10 batches of ED runs, the final global system resistance rose from 15.8 Ω (1st batch) to 43.9 Ω (10th batch), and the treatment ending time was delayed from 120 min (1st batch) to 162 min (10th batch). The initial value of the global system resistance corresponds to the intrinsic resistance to solution as well as the other compartments of the ED stack such as membranes, spacers, and electrode plates. Meanwhile, ion migration kinetics and the fouling nature of ion-exchange membranes highly restrict the final value of the global system resistance [20,21]. As all the initial experiment parameters are constant for each batch of the ED process, it can be inferred that the membrane fouling caused the rapid elevation of the global system resistance and the gradually delayed treatment ending time. 

#### 3.1.2. Deacidification Ratio

In this work, the ED apparatus was designed to remove acid from CAH. Hence, the deacidification ratio is an essential index for evaluating the ED’s operating condition. As shown in Figure 2c, the deacidification rate was plotted with time for 10 batches. More than 99% of acid was removed from CAH at the end of each batch of the ED process. However, the instantaneous deacidification rate gradually declined with the repeated batch running. Figure 2e shows the variation tendency of the instantaneous deacidification rate. Along with the repeated batch running, the deacidification ratio declined from 83.0% (1st batch) to 72.5% (10th batch) at the 72nd min, and from 99.0% (1st batch) to 96.1% (10th batch) at the 120th min, respectively. This decreased performance could be explained by a progressive fouling of the ion-exchange membranes, causing the decline of H^+^ permeate flux and deacidification ratio [20,22].

#### 3.1.3. H^+^ Relative Energy Consumption

Energy consumption is an essential index for evaluating process efficiency. As the aim of this work is acid recovery, H^+^ relative energy consumption was analyzed in the repeated batch. The H^+^ relative energy consumption is dependent on some factors, such as target ion concentration (H^+^), co-ion effect (Na^+^, K^+^, etc.), applied voltage, pH, flow rate, foulants, and other factors [23]. In a single batch, as shown in Figure 2d, the H^+^ relative energy consumption increases instantly with the extension of time. At the beginning of each batch experiment, as the high concentrations of H^+^ and SO_4_^2−^ in CAH could reduce the electrical resistance on the membrane surfaces, the mobility of H^+^ ions is fast. As a result, the ED process is operating with higher current efficiency and lower energy consumption. With the ED experiment running, the concentration of H^+^ and SO_4_^2−^ in CAH decreased, and mass transfer resistance arose. The competitive advantage of co-ions (Na^+^, K^+^, etc.) increases gradually. Due to the variation in migration rates, the co-ion transfer is accelerated [24]. Meanwhile, as the concentration of H^+^ and SO_4_^2−^ in the concentrate solution arises, the reverse ion leakage (back diffusion of ion from concentrate to dilute compartment) is increased gradually. All of these factors result in an increase in H^+^ relative energy consumption [25].

With the repeated batch running, as shown in Figure 2f, the instantaneous H^+^ relative energy consumption increases gradually. For instance, the H^+^ relative energy consumption increased from 0.07 Kw·h/mol (1st batch) to 0.56 Kw·h/mol% (10th batch) at the 36th min, and from 1.07 Kw·h/mol (1st batch) to 1.40 Kw·h/mol (10th batch) at the 90th min, respectively. The increased H+ relative energy consumption could also be explained by a progressive fouling of the ion-exchange membranes. 

In short conclusion: (1) More than 99% of acid can be removed from CAH in this work. (2) A non-negligible membrane fouling occurred during the ED process and was aggravated by the repeated batch running. (3) The performance of this ED apparatus declined, and correspondingly, the energy consumption increased.

### 3.2. Analysis of CAH Component

The CAH specifications before and after the ED process are listed in Table 4. The ED process affected the CAH properties. Throughout the ED process, H^+^ and SO_4_^2−^ ions migrated from the dilute to the concentrate compartment. As a result, the pH of the CAH liquor rose from 1.24 to 3.29, and more than 99% of acid was removed. The conductivity of CAH decreased from 18.04 mS/cm to 1.05 mS/cm. Besides H^+^ and SO_4_^2-^ ions, the migration of co-ions (Na^+^, K^+^, etc.) contributed to the decrement of conductivity. Especially in the last part of the ED process, as the variation in the migration rate increased, a greater proportion of co-ions migrated from the dilute to the concentrate compartment [24]. 

**Figure 3:**
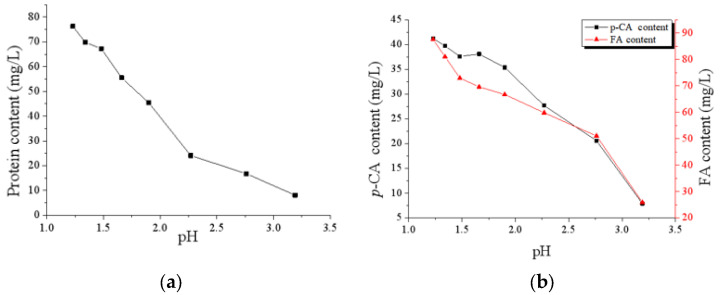
Evaluation of protein and phenolic acids contents of CAH during ED deacidification process ((**a**): protein; (**b**): phenolic acids).

Notably, the FA, *p*-CA, and protein contents of CAH liquor decreased during the ED process (Figure 3). After the ED process, about 90% of protein, 70% of FA, and 80% of *p*-CA disappeared from the CAH liquor. As the CEM and AEM membranes are dense and compact, it is hardly possible for them to migrate to the concentrate compartment. According to the mass conservation law, there are only two possibilities: (a) precipitation from the CAH liquor; (b) adsorption to the surface of membranes.

### 3.3. The ζ-Potential Variations of CAH

**Figure 4:**
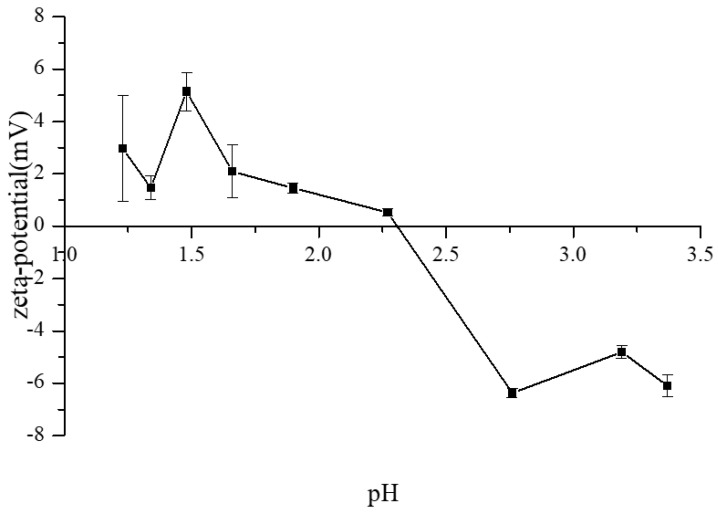
Evaluation of zeta potential as a function of pH for CAH liquor during ED deacidification process.

It is the basic principle of colloidal chemistry that an electrical double layer (the Helmholtz layer and an extended diffuse layer) around a charged surface reflects the specific properties of the counter-ions and the nature of the colloidal interface [26]. The zeta potential of colloidal particles is an indicator of the surface charge, which is estimated based on the electrophoretic mobility in the electric field and represents the stability of the colloidal particles or interfaces [27]. Hence, the zeta potential of colloidal particles is an important factor affecting electrodialysis performance and provides valuable information for the prediction of fouling potentials. The electrokinetic properties of colloidal particles are affected by the solution pH, which is observed in electrophoretic mobility measurements. As the deacidification proceeded, the pH increased. The zeta potentials of CAH as a function of pH are presented in Figure 4. At the beginning of the ED process, the CAH is pellucid. The initial value of zeta potential is about 3 mV, which means the surface charge of colloidal particles in CAH is weakly positive. The colloidal particles of CAH are relatively stable, as the electrical double-layer repulsion between the colloidal particles avoids particle aggregation. With the run of the ED process, the acid (H+ and corresponding anion) was removed, and the pH was increased from 1.1 to 3.4. The zeta potential of colloidal particles in CAH changed from a positive value to a negative value and reached the isoelectric point (IEP) at pH 2.3, approximately. During this ED process, the proton (H^+^) on the surface of colloidal particles is successively desorbed and removed, which leads to electrical double-layer repulsion and particle aggregation. The CAH liquor loses its colloidal stability and becomes cloudy.

Based on the zeta potential analysis, it can be concluded that some colloidal particles, which have a positively charged surface, become unstable, and isoelectric point precipitation of colloidal particles occurs during the ED process. The aggregation of colloidal particles depends on several factors, including temperature, electrolyte concentration, and pH. For commercially available softwood kraft lignin in diluted alkaline solutions, the electrokinetic properties of lignin particles are affected by the solution pH values [28]. The components of precipitates are mainly lignin and protein; their colloidal stability is possibly decreased with deprotonation. This will be discussed further in the next section.

### 3.4. Membrane Surface Analysis for Fouling Identification

**Figure 5:**
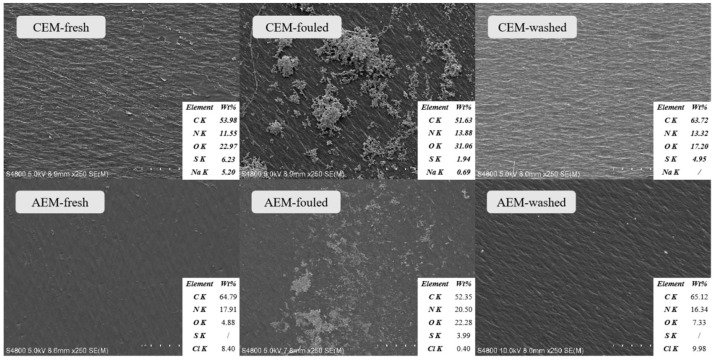
The SEM electron micrographs with EDS of CEM and AEM membranes in three states (fresh, fouled, and washed).

The morphology of the membrane’s surface was analyzed by means of microscopic observation. SEM images of CEM and AEM in three states (fresh, fouled, and washed) are shown in Figure 5, along with the corresponding EDS. The sides of the CEM and AEM surfaces that are directly contacted with the CAH liquor were fouled. Only these sides were measured. The other sides of both membranes that are contacted with the concentrate presented a clean surface and had no obvious fouling or alteration. Both the fresh CEM and AEM membranes presented a clean surface in the image at 250× magnification. By contrast, after the ED process, a noticeable deposition layer was formed on the surface of both membranes. After the cleaning process, the deposited layer has been removed thoroughly, shown as a clean membrane.

In addition, from the EDS analysis, compared with the fresh CEM, even more oxygen and nitrogen constituted the fouling layer of the CEM. After the ED process, the O/C ratios of the CEM fouling layer increased from 43% to 60%, and the N/C ratios of the CEM fouling layer increased from 21% to 27%. After the cleaning process, the ratios fall back correspondingly. Similar to the AEM, after the ED process, the O/C ratios of the AEM fouling layer increased from 8% to 43%, and the N/C ratios of the AEM fouling layer increased from 28% to 39%. After the cleaning process, the ratios fall back correspondingly. Thereby, it can be presumed that this membrane was slightly affected by the fouling phenomenon.

### 3.5. Analysis of Membrane Foulant

To identify the chemical components of the membrane foulant, the classical Soxhlet extraction was performed with pure acetone as the extractant (liquid-to-solid ratio = 100:1; operating temperature: 62 °C; operating time: 24 h). Subsequently, the extract and raffinate of membrane foulant were freeze-dried to remove the solvent.

**Figure 6:**
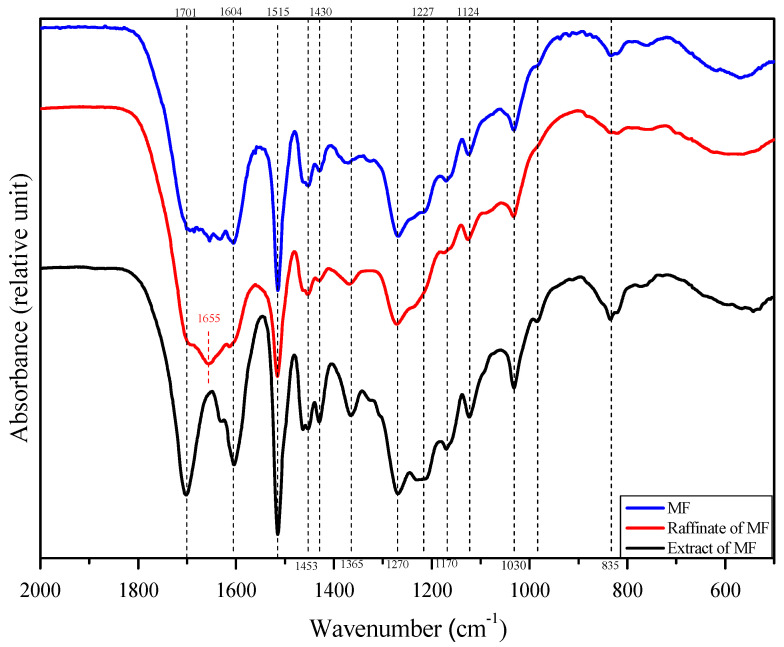
The FTIR spectra of membrane foulant.

FTIR was used to contrastively analyze the three samples (a: original membrane foulant; b: raffinate of membrane foulant; and c: extract of membrane foulant), and the spectra are shown in Figure 6 and Table 5. All samples show the typical spectral bands (1604, 1515, 1453, 1430, and 1270 cm^−1^) that represented the aromatic regions of lignin, which indicates the predominance of lignin in membrane foulant [29]. Compared with the original membrane foulant (A), the intensity of the corresponding lignin signals of extract (C) is significantly increased, and the corresponding value of raffinate (B) is slightly decreased. This could be explained by the fact that the lignin could be dissolved by pure acetone in the Soxhlet extraction process, but the protein hydrolysate is insoluble in pure acetone. Therefore, a major part of the lignin was transferred to the extract (C). There are no other lignin signals in the spectra of the extract (C). And in the spectra of raffinate (B), the intensity of the spectral band at 1655 cm^−1^, which is assigned to the α-helix (amide I peak) structure of proteins, increased abruptly [30]. This proves the existence of proteins in the membrane foulant. This spectral change was due to the fact that the removal of lignin raised the content of proteins in the membrane foulant. The amide I peak is the most prominent peak in the protein spectrum and can be attributed to the α-helical component of the secondary structure [31]. Noticeably, after the long Soxhlet extraction, the lignin signals are still dominant in the spectra of raffinate (B). It may be concluded that strong chemical bonding, such as covalent linkage, excited between the residual lignin and protein. It has been reported that lignin could cross-couple with the side chain of amino acids, thereby creating covalent bonds between lignin and proteins [32].

Furthermore, the component that has been transferred to extract (C) was inferred to be a mixture of protein and residual lignin with weakly interactions. During the acid hydrolysis process, the predominant reaction of lignin is the cleavage of the β-aryl ether band, which is the most abundant interunit linkage in lignin. The cleavage of the β-aryl ether band significantly decreases the mean molecular weight of lignin and increases its solubility as well as the content of phenolic hydroxyls in the acid hydrolysate. The soluble lignin can produce adsorption sites for protein through hydrophobic, electrostatic, and hydrogen bonding interactions.

Moreover, the spectral bands at 1701, 1170 cm^−1^ are assigned to the C=O in ester groups; the spectral band at 1032 cm^−1^ is assigned to the glycosidic linkages (C-O-C) stretching, implying that the saccharides and lignin co-existed [33]. Combined with the conclusion of HSQC, it can be concluded that the LCCs are one main form of lignin in membrane foulant, which is consistent with the current literature [34]. However, the direct assessment of structural changes at the molecular level of membrane foulant cannot be achieved only by using FTIR spectra because of the heterogeneous nature of plant materials and the complicated reaction process.

**Figure 7:**
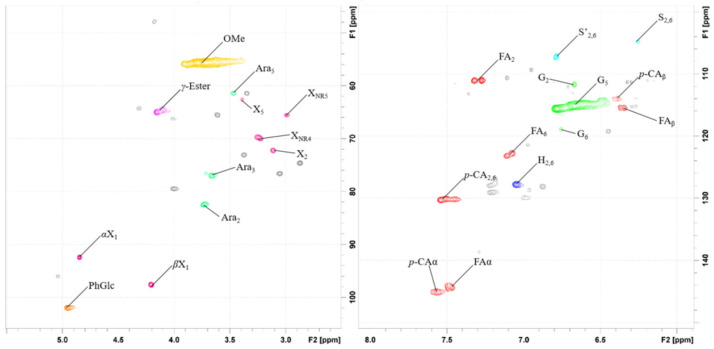
The 2D-HSQC NMR spectra of membrane foulant. The cross-signals assigned in the NMR spectra are listed in Table S2 of the Supplementary Material.

The HSQC technique provided important information about the structural characteristics and the distribution of chemical linkages in the membrane foulant [35]. The HSQC spectra of the membrane foulant are presented in Figure 7. The main assigned peaks are illustrated in Table 6 by comparison with literature data [36], and the main substructures are depicted in Figure 7. We can clearly distinguish the signals of methoxyls groups (-OCH_3_, *δ*_C_/*δ*_H_ 55.7/3.74), guaiacyl units (G, *δ*_C_/*δ*_H_: G_2_ 111.9/6.68, G5 115.1/6.67, G6 119.0/6.68), *p*-hydroxyphenyl units (H, *δ*_C_/*δ*_H_: H_2,6_ 127.9/7.05), *p*-coumarate (*p*-CA, *δ*_C_/*δ*_H_: *p*-CA_2, 6_ 130.3/7.54, *p*-CA*_a_* 145.1/7.57, *p*-Ca_β_ 113.8/6.29) and ferulate (FA, *δ_C_/δ_H_*: FA_2_ 111.2/7.33, FA_6_ 123.2/7.12, FA_a_ 145.1/7.57) as their C*_a_*-H*_a_* correlation signals, respectively. The structures of lignin composition are shown in Figure S1. The methoxyl groups coupling with aromatic groups are a useful diagnostic feature in identifying substructures of lignin. During the lignin biosynthesis process, lignin forms a variety of linkages with carbohydrates (cellulose and hemicellulose). The main types of native LCC linkages are believed to be phenol glycoside linkages (PhGlc), benzyl esters linkages (γ-Ester), and benzyl ethers linkages (BE) (Figure S1) [37]. Three typical linkages of LCC have been observed, including phenol glycoside (PhGlc, *δ*_C_/*δ*_H_:102.1/4.96) linkages, γ-Ester (Est, *δ*_C_/*δ*_H_:65.1/4.16) linkages, and carbohydrate structures (α-(1→3)-L-arabinofuranoside, *δ_C_/δ_H_*: Ara_3_ 77.1/3.66, Ara_2_ 82.4/3.73) in the HSQC spectra. Notably, the signals of benzyl ethers (BE) disappeared, suggesting that benzyl ether linkages (BE) were mostly cleaved during the hydrolysis process. This is the first time that the complex hydrolysis process has been revealed. Moreover, various signals from the associated carbohydrates could also be found in the HSQC spectra. The absence of inter-units indicates that membrane foulant was only a mixture of small molecules (lignin) rather than a polymer complex, which is consistent with the GPC results (see below).

After the removal of the protein, the molecular weight of the foulant was also analyzed using GPC (Figure S2). The *Mn* of the foulant is 798 Da in DMSO. Considering the molecular weight scale of carbohydrate monomers (glucose: 180.16) and phenolic acid monomers (ferulic acid: 194.18), the membrane foulants are possibly LCCs with a small molecular weight.

Lemaire et al. performed ED deacidification to purify pentose and adapted an ultrafiltration unit to remove macromolecules such as protein and lignin [12,13]. In this work, we confirmed the molecular structure and molecular weight of membrane foulant for the first time. We suggested that the fouling could be mitigated when the pH value is controlled below the IEP. 

### 3.6. Proposed Fouling Mechanisms

**Figure 8:**
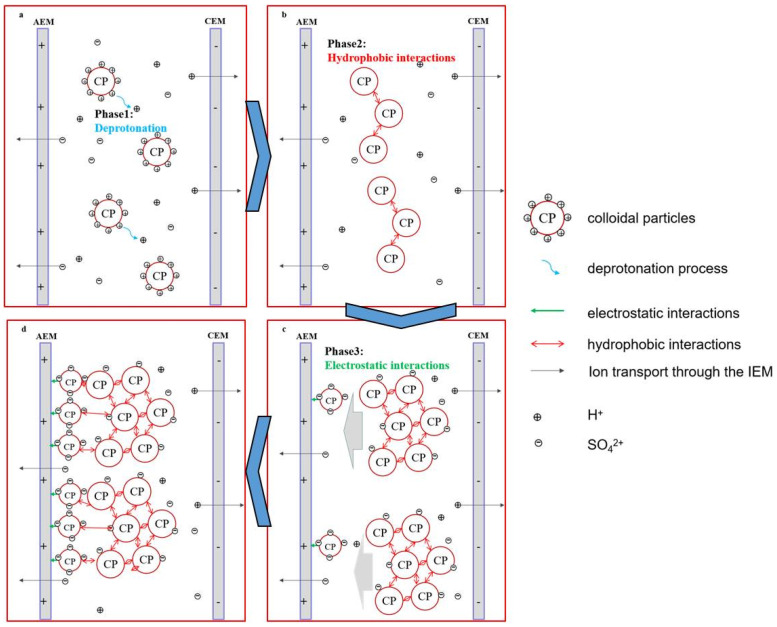
Proposed fouling mechanism of AEM and CEM during electrodialysis deacidification. (**a**) deacidification; (**b**) hydrophobic interactions; (**c**) electrostatic interactions; (**d**) deterioration.

According to the FTIR and HSQC analyses of membrane foulant, combined with the zeta potential result of CAH, a postulated membrane fouling mechanism is put forward in our work (Figure 8). It is well known that the stability properties of colloidal particles are affected by the solution pH values. As discussed in Section 3.3, the zeta potential of colloidal particles is an indicator of the surface charge, which is estimated based on the electrophoretic mobility in the electric field and represents the stability of the colloidal particles. During the whole deacidification process, the sulfuric acid in CAH was transported to the concentrate compartments. As a result, the pH was continuously increased from 1.1 to 3.4, the zeta potential of colloidal particles in CAH changed from a positive value to a negative value, and it reached the isoelectric point (IEP) at pH 2.3. Therefore, it can be divided into four stages.

At the initial stage (deacidification), it is mainly the acid transport process. The proton (H^+^) ions were transported from CEM, while the corresponding anions (SO_4_^2+^ and so on) were transported from AEM. Subsequently, with the loss of proton ions in the bulk solution, the proton (H^+^) ions on the charged surface of colloidal particles were dissociated into the solution [12,13]. As the zeta potential approaches zero, the colloidal stability of the colloid particles gradually decreases, as shown in Figure 8a. 

At the second stage (hydrophobic interactions), the zeta potential reaches zero, and the double layer of colloidal particles is compressed down to the threshold. Due to the inter-molecular hydrophobic interactions, the repulsive forces between the colloidal particles reduce and attractive forces become dominant, resulting in the self-aggregation of the colloidal particles, as shown in Figure 8b [18,28,38]. 

At the third stage (electrostatic interactions), with the further increase in pH, the zeta potential of colloidal particles in CAH changes to a negative value. The surface charge of colloidal particles becomes negative. According to the above-mentioned FTIR and HSQC analyses of membrane foulants, they are rich in carboxylic and phenolic groups (in the form of FA and *p*-CA). Part of them become gradually dissociated at the higher pH level. This is supposed to be one of the crucial reasons that the zeta potential changes to a negative value. The negatively charged colloidal particles near the AEM began to precipitate at the surface due to the electrostatic interactions, as shown in Figure 8c.

**Figure 9:**
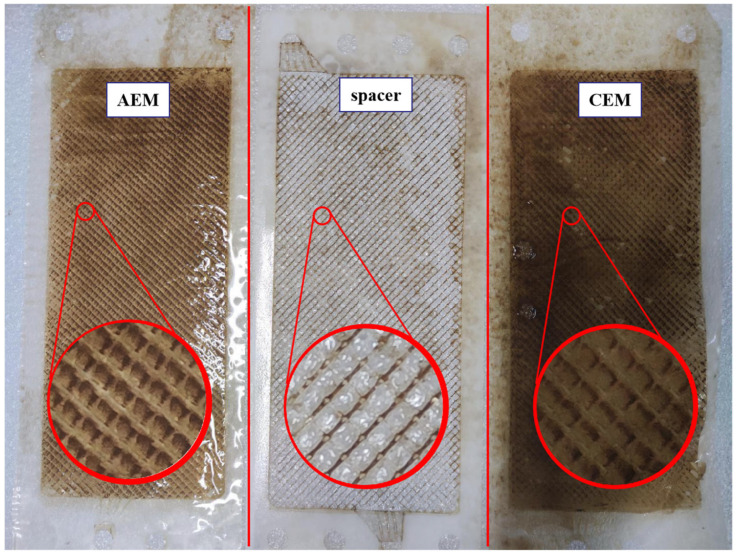
Photograph of fouled membrane components.

At the fourth stage (deterioration), with the further increase in pH, the zeta potential drops slowly. In the electric field, all the negatively charged colloidal particles move towards the AEM, and the self-aggregation deteriorates further. The colloidal nuclei develop in size and number, and the deposits that form on the AEM surface severely obstruct the exchange interface of ions, and ultimately the whole room of dilute compartments is jammed by the colloidal nuclei, which adhere to the surfaces of spacers and CEM, as shown in Figure 8c. It can be proven by the photograph of fouled membrane components (Figure 9). Meanwhile, the performance of this ED apparatus declined, and correspondingly, energy consumption increased seriously.

## 4. Conclusions

The selected commercial IEMs show excellent performance in this ED process; a deacidification ratio of 99% has been realized. At the same time, membrane fouling occurred during the ED process and aggravated with the repeated batch running.

For the first time, we observed the formation of deposit layers on the surfaces of both anion and cation exchange membranes, which were in direct contact with the acid hydrolysate solution during the ED process. The membrane foulants are mostly composed of hydrolysates of protein and lignin. 

For the first time, the LCCs were reported as membrane foulants in the ED process. We found that: (1) the lignin component of the membrane foulant is in the form of LCCs; (2) *p*-CA and FA are cross-linked with carbohydrate through the phenol glycoside (PhGlc) linkages and γ-Ester (Est) linkages; (3) a strong chemical bonding, possibly a covalent linkage, existed between the lignin and protein in the foulant; and (4) during the ED process, the zeta potential of colloidal particles in CAH changed from a positive value to a negative value, and an IEP around pH 2.3 was reached. 

Based on the nature of membrane foulant, a proposed fouling mechanism for this ED process was discussed in this work. According to the fouling mechanisms, throughout the ED process, the pH of the CAH gradually increased, which decreased the solubility of colloidal particles. Destabilized colloidal particles started to self-aggregate and form deposits on the membrane surface (firstly AEM). Over time, these deposits covered the surface of AEM, and ultimately the whole room of dilute compartments, including the surface of CEM, was jammed by the colloidal nuclei.

In the end, a suggestion should be given to the lignocellulose biorefinery industry: the endpoint of the deacidification process should be lower than the IEP, aiming to avoid serious membrane fouling. Moreover, the post-treatment method (flocculation and ultrafiltration technology) can be tested to remove the colloidal particles. Based on the understanding of fouling mechanisms, an integrated ED process, combined with flocculation and ultrafiltration technology, will be designed in future work.

## Figures and Tables

**Table 1:** Physic-chemical properties and composition of the CAH feedstock.

Item	Value	Unit
Brix	7.8	%
pH	1.1	–
Conductivity	17.24	mS/cm
OD420	2.2	–
Glucose	3.57	g/L
Xylose	47.53	
Arabinose	4.26	
Sodium	355.0	mg/L
Potassium	473.8	
Magnesium	39.4	
Calcium	98.5	
Chlorure	159.4	
Sulfate	3935.7	

**Table 2:** The main characteristics of AEM and CEM used in this study.

Membrane Type	AEM	CEM
Membrane code	TWEDA1	TWEDC1
Thickness/wet (µm)	40–50	40–50
IEC ^a^ (ion exchange capacity, mmol/g)	0.90–1.10	0.90–1.10
Area resistance ^b^ (Ω·cm^2^)	≤2.5	≤3.3
Water uptake ^c^ (%)	15–20	15–20
Transport number ^d^	≥0.98	≥0.97

The data were collected from the product brochure provided by manufacturers. ^a^ Ion exchange capacity test conditions: relative to the dry membranes, 25 °C; ^b^ area resistance of AEM and CEM was measured as Cl^−^ and Na^+^ form in 0.5 mol/L NaCl at 25 °C, respectively; ^c^ water uptake was determined by weight ratio of absorbed water to membrane dry weight; ^d^ transport number of AEM and CEM was measured as Cl^−^ and Na^+^ form in 0.5 mol/L and 0.1 mol/L NaCl solution at 25 °C, respectively.

**Table 3:** Applied operational conditions during electrochemical deacidification method.

Operational Conditions	Data
Number of operating units	4
Re-circulation flow rate	0.7 L/min
Applied current density	64.33 A/m^2^
Effective membrane surface area	0.0187 m^2^
Initial volume of each solution	2 L
Operational temperature	25 °C
Initial Na_2_SO_4_ concentration	0.1 mol/L
Initial pure water	≥18.2 MΩ·cm

**Table 4:** Characteristics of the examined CAH liquor.

Characteristics	Before *ED*	After *ED*
pH	1.24 ± 0.01	3.29 ± 0.06
Conductivity(mS/cm)	18.04 ± 0.46	1.05 ± 0.04
Protein (mg/L) *	76.26	8.09
FA (mg/L) *	87.52	25.92
*p*-CA (mg/L) *	41.24	7.91

* The samples of 2nd batch experiment were chosen for the analysis of protein and phenolic acid contents.

**Table 5:** Peak assignment of FTIR spectra of membrane foulant.

Maxima at cm^−1^, Absorbance	Chemical Group	Band Assignment
835	S units	C–H out of plane at positions 2 and 6 of in etherified syringyl units (S units)
1032	Glucosidic bonds	C–O–C stretching
1170	C=O in ester groups of lignin units	S ring+G ring condensed (G ring substituted at position 5) C–C, C–O, C=O stretching; G condensed>G etherfied; aromatic C–H in-plane deformation; typical for G units; primary OH
1227	C-C, C-O, and C=O bonds	S ring + G ring condensed (G ring substituted at position 5) C–C, C–O, C=O stretch; G condensed>G etherfied; aromatic C–H in-plane deformation; typical for G units; primary OH
1270	C=O bond	C=O stretching
1365	Aliphatic C–H in CH_3_ not in OCH_3_; phenolic OH	aliphatic C–H stretching in CH_3_ not in OCH_3_; phenolic O–H stretching
1430	Aromatic skeletal	aromatic skeletal vibrations combined with C–H in-plane deformation
1463	Aromatic skeletal	C–H deformation
1515	Aromatic skeletal	aromatic skeletal vibrations G>S
1604	Aromatic skeletal	vibration of aromatic skeletal; C6-point double bond O stretch; S>G, G condensed>G etherified
1655	Amide I peak	α-helix
1701	Ester group	C=O stretching
2840	Methylene group	C–H stretching
2938	Methyl group	

**Table 6:** Peak assignment of HSQC spectra of membrane foulant.

Labels	δ_C_/δ_H_	Assignment
-OCH_3_	55.7/3.74	C–H in methoxyls
γ-Ester	65.1/4.16	γ-Ester linkages in LCC
Ara_3_	77.1/3.66	C_3_–H_3_ in *α*-(1→ 3)-L-arabinofuranoside
D_β_	79.6/4.01	Cβ’-Hβ’ in spirodienone substructures(D)
Ara_2_	82.4/3.73	C_2_–H_2_ in *α*-(1 → 3)-L-arabinofuranoside
PhGlc	102.1/4.96	Phenyl glycoside linkages in LCC
S_2, 6_	104.9/6.26	C2, 6-H2,6 in etherified syringyl units (S)
S’_2, 6_	107.2/6.79	C2, 6-H2,6 in etherified syringyl units (S’)
FA_2_	111.2/7.33	C2–H2 in ferulate (FA)
G_2_	111.9/6.68	C2–H2 in guaiacyl units (G)
*p*-CA_β_	113.8/6.29	C_β_–H_β_ in *p*-coumarate (*p*-CA)
G_5_	115.1/6.67	C5–H5 in guaiacyl units (G)
G_6_	119.0/6.68	C6–H6 in guaiacyl units (G)
FA_6_	123.2/7.12	C6–H6 in ferulate (FA)
H_2,6_	127.9/7.05	C2, 6-H2,6 in *p*-hydroxyphenyl units (H)
*p*-CA_2, 6_	130.3/7.54	C2, 6-H2,6 in *p*-hydroxyphenyl units (H)
*p*-CA*_a_*, FA*_a_*	145.1/7.57	C*a*-H*a* in *p*-coumarate (*p*-CA) and ferulate (FA)

## Data Availability

The article does not contain new data.

## Supplementary Materials

The following supporting information can be downloaded at: https://www.mdpi.com/article/10.3390/membranes13030256/s1, Figure S1. H, G, S, p-CA and FA composition in the lignin polymer, and linkages between lignin and carbohydrate including PhGlc and γ-Ester.p-hydroxyphenyl units (H); guaiacyl units (G); etherified syringyl units (S); p-coumarate (p-CA); ferulate (FA); phenol glycoside (PhGlc); benzyl esters linkages (γ-Ester); Figure S2. Measurement of molecular weights of membrane foulant by gel permeation chromatography (GPC). The eluent was DMSO. Detection was achieved with a Knauer differential refractive index detector (RID).

## References

[1] Ragauskas A.J., Beckham G.T., Biddy M.J., Chandra R., Chen F., Davis M.F., Davison B.H., Dixon R.A., Gilna P., Keller M. (2014). Lignin Valorization: Improving Lignin Processing in the Biorefinery. Science.

[2] Shen X.J., Sun R.C. (2021). Recent advances in lignocellulose prior-fractionation for biomaterials, biochemicals, and bioenergy. Carbohydr. Polym..

[3] Maki-Arvela P., Salmi T., Holmbom B., Willfor S., Murzin D.Y. (2011). Synthesis of Sugars by Hydrolysis of Hemicelluloses—A Review. Chem. Rev..

[4] Davison B.H., Drescher S.R., Tuskan G.A., Davis M.F., Nghiem N.P. (2006). Variation of S/G ratio and lignin content in a Populus family influences the release of xylose by dilute acid hydrolysis. Appl. Biochem. Biotechnol..

[5] Lavarack B.P., Griffin G.J., Rodman D. (2002). The acid hydrolysis of sugarcane bagasse hemicellulose to produce xylose, arabinose, glucose and other products. Biomass Bioenergy.

[6] Chen X.Q., Yang Q.L., Si C.L., Wang Z.J., Huo D., Hong Y.M., Li Z.Q. (2016). Recovery of Oligosaccharides from Prehydrolysis Liquors of Poplar by Microfiltration/Ultrafiltration Membranes and Anion Exchange Resin. ACS Sustain. Chem. Eng..

[7] Wang X.J., Zhuang J.S., Fu Y.J., Tian G.Y., Wang Z.J., Qin M.H. (2016). Separation of hemicellulose-derived saccharides from wood hydrolysate by lime and ion exchange resin. Bioresour. Technol..

[8] Wang X.J., Zhuang J.S., Jiang J.G., Fu Y.J., Qin M.H., Wang Z.J. (2015). Separation and purification of hemicellulose-derived saccharides from wood hydrolysate by combined process. Bioresour. Technol..

[9] Wang Z.J., Wang X.J., Fu Y.J., Li Z.Q., Zhang F.S., Qin M.H. (2015). Colloidal behaviors of lignin contaminants: Destabilization and elimination for oligosaccharides separation from wood hydrolysate. Sep. Purif. Technol..

[10] Waheed H., Farrukh S., Hussain A., Mukhtar A., Mubashir M., Saqib S., Ullah S., Peter A.P., Khoo K.S., Show P.L. (2022). Green synthesized nano-cellulose polyethylene imine-based biological membrane. Food Chem. Toxicol..

[11] Jamil A., Ching O.P., Iqbal T., Rafiq S., Zia-ul-Haq M., Shahid M.Z., Mubashir M., Manickam S., Show P.L. (2021). Development of an extended model for the permeation of environmentally hazardous CO_2_ gas across asymmetric hollow fiber composite membranes. J. Hazard. Mater..

[12] Blanc C.-L., Lemaire J., Duval F., Théoleyre M.-A., Pareau D. (2017). Purification of pentoses from hemicellulosic hydrolysates without neutralization for sulfuric acid recovery. Sep. Purif. Technol..

[13] Lemaire J., Blanc C.-L., Duval F., Théoleyre M.-A., Pareau D. (2016). Purification of pentoses from hemicellulosic hydrolysates with sulfuric acid recovery by using electrodialysis. Sep. Purif. Technol..

[14] Pismenskaya N., Bdiri M., Sarapulova V., Kozmai A., Fouilloux J., Baklouti L., Larchet C., Renard E., Dammak L. (2021). A Review on Ion-Exchange Membranes Fouling during Electrodialysis Process in Food Industry, Part 2: Influence on Transport Properties and Electrochemical Characteristics, Cleaning and Its Consequences. Membranes.

[15] Dammak L., Fouilloux J., Bdiri M., Larchet C., Renard E., Baklouti L., Sarapulova V., Kozmai A., Pismenskaya N. (2021). A Review on Ion-Exchange Membrane Fouling during the Electrodialysis Process in the Food Industry, Part 1: Types, Effects, Characterization Methods, Fouling Mechanisms and Interactions. Membranes.

[16] Mikhaylin S., Bazinet L. (2016). Fouling on ion-exchange membranes: Classification, characterization and strategies of prevention and control. Adv. Colloid. Interfac..

[17] Haddad M., Bazinet L., Savadogo O., Paris J. (2017). Electrochemical acidification of Kraft black liquor: Impacts of pulsed electric field application on bipolar membrane colloidal fouling and process. J. Membr. Sci..

[18] Haddad M., Mikhaylin S., Bazinet L., Savadogo O., Paris J. (2017). Electrochemical acidification of Kraft black liquor by electrodialysis with bipolar membrane: Ion exchange membrane fouling identification and mechanisms. J. Colloid Interface Sci..

[19] Xu T., Huang C. (2008). Electrodialysis-based separation technologies: A critical review. AIChE J..

[20] Casademont C., Farias M.A., Pourcelly G., Bazinet L. (2008). Impact of electrodialytic parameters on cation migration kinetics and fouling nature of ion-exchange membranes during treatment of solutions with different magnesium/calcium ratios. J. Membr. Sci..

[21] Casademont C., Sistat P., Ruiz B., Pourcelly G., Bazinet L. (2009). Electrodialysis of model salt solution containing whey proteins: Enhancement by pulsed electric field and modified cell configuration. J. Membr. Sci..

[22] Lee H.J., Moon S.H. (2004). Fouling mitigation in the repeated batch runs of electrodialysis with humate foulant. Korean J. Chem. Eng..

[23] Afifah D.N., Ariyanto T., Supranto S., Prasetyo I. (2018). Separation of Lithium Ion from Lithium-Cobalt Mixture using Electrodialysis Monovalent Membrane. Eng. J..

[24] Gregor H.P., Peterson M.A. (1964). Electrodialytic Polarization of Ion-Exchange Membrane Systems. J. Phys. Chem..

[25] Parsa N., Moheb A., Mehrabani-Zeinabad A., Masigol M.A. (2015). Recovery of lithium ions from sodium-contaminated lithium bromide solution by using electrodialysis process. Chem. Eng. Res. Des..

[26] Suwal S., Doyen A., Bazinet L. (2015). Characterization of protein, peptide and amino acid fouling on ion-exchange and filtration membranes: Review of current and recently developed methods. J. Membr. Sci..

[27] Lee H.-J., Hong M.-K., Han S.-D., Cho S.-H., Moon S.-H. (2009). Fouling of an anion exchange membrane in the electrodialysis desalination process in the presence of organic foulants. Desalination.

[28] Norgren M., Edlund H., Wågberg L., Lindström B., Annergren G. (2001). Aggregation of kraft lignin derivatives under conditions relevant to the process, part I: Phase behaviour. Colloids Surf. A.

[29] Derkacheva O., Sukhov D. (2008). Investigation of Lignins by FTIR Spectroscopy. Macromol. Symp..

[30] Moghaddam L., Rencoret J., Maliger V.R., Rackemann D.W., Harrison M.D., Gutiérrez A., del Río J.C., Doherty W.O.S. (2017). Structural Characteristics of Bagasse Furfural Residue and Its Lignin Component. An NMR, Py-GC/MS, and FTIR Study. ACS Sustain. Chem. Eng..

[31] De Ninno A., Castellano A.C. (2011). Deprotonation of Glutamic Acid Induced by Weak Magnetic Field: An FTIR-ATR Study. Bioelectromagnetics.

[32] Mnich E., Bjarnholt N., Eudes A., Harholt J., Holland C., Jørgensen B., Larsen F.H., Liu M., Manat R., Meyer A.S. (2020). Phenolic cross-links: Building and de-constructing the plant cell wall. Nat. Prod. Rep..

[33] Hood C., Laredo T., Marangoni A.G., Pensini E. (2021). Water-repellent films from corn protein and tomato cutin. J. Appl. Polym. Sci..

[34] Al-Rudainy B., Galbe M., Wallberg O. (2017). Influence of prefiltration on membrane performance during isolation of lignin-carbohydrate complexes from spent sulfite liquor. Sep. Purif. Technol..

[35] Yuan T.-Q., Sun S.-N., Xu F., Sun R.-C. (2011). Characterization of Lignin Structures and Lignin–Carbohydrate Complex (LCC) Linkages by Quantitative 13C and 2D HSQC NMR Spectroscopy. J. Agric. Food. Chem..

[36] Wen J.-L., Xue B.-L., Xu F., Sun R.-C., Pinkert A. (2013). Unmasking the structural features and property of lignin from bamboo. Ind. Crops Prod..

[37] Balakshin M., Capanema E., Gracz H., Chang H.-M., Jameel H. (2011). Quantification of lignin–carbohydrate linkages with high-resolution NMR spectroscopy. Planta.

[38] Norgren M., Edlund H., Wågberg L. (2002). Aggregation of Lignin Derivatives under Alkaline Conditions. Kinetics and Aggregate Structure. Langmuir.

