# Supplementary material for: Electrodialysis Deacidification of Acid Hydrolysate in Hemicellulose Saccharification Process: Membrane Fouling Identification and Mechanisms
Source: Membranes (Basel). 2023 Feb 21;13(3):256. doi: 10.3390/membranes13030256 (PMC10053187; doi:10.3390/membranes13030256)
Supplement: Supplementary file 1 [file membranes-13-00256-s001.zip › membranes-2177485-supplementary.pdf]

Electrodialysis deacidification of acid hydrolysate in hemicellulose saccharification process: membrane fouling identification and mechanisms

Xitao Luo[1,2], Lingling Sun[1], Qinghui Shou[1,2], Xiangfeng Liang[1,2,*], Huizhou Liu[1,2,*]

1 CAS Key Laboratory of Bio-Based Materials, Qingdao Institute of Bioenergy and Bioprocess Technology (QIBEBT), Chinese Academy of Sciences(CAS), Qingdao 266101, China

2 University of Chinese Academy of Sciences, Beijing 100049, P.R. China

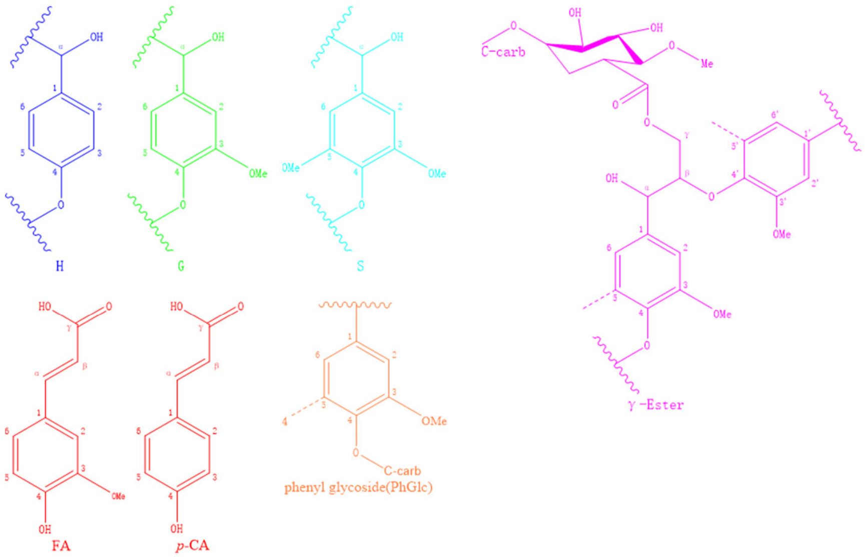


**Figure S1.** H, G, S, *p*-CA and FA composition in the lignin polymer, and linkages between lignin and carbohydrate including PhGlc and γ-Ester.

*p*-hydroxyphenyl units (H); guaiacyl units (G); etherified syringyl units (S); *p*-coumarate (*p*-CA); ferulate (FA); phenol glycoside (PhGlc); benzyl esters linkages (γ-Ester)

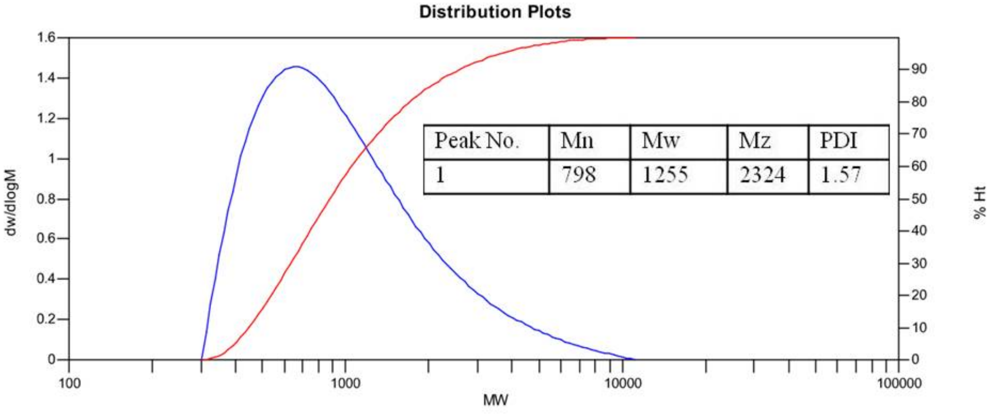


| Peak No. | Mn | Mw | Mz | PDI |
|---|---|---|---|---|
| 1 | 798 | 1255 | 2324 | 1.57 |

**Figure S2.** Measurement of molecular weights of membrane foulant by gel permeation chromatography (GPC). The eluent was DMSO. Detection was achieved with a Knauer differential refractive index detector (RID).
